# Fabrication of glass/madar fibers reinforced hybrid epoxy composite: a comprehensive study on the material stability

**DOI:** 10.1038/s41598-024-53178-x

**Published:** 2024-04-10

**Authors:** Thandavamoorthy Raja, D. Yuvarajan, Saheb Ali, G. Dhanraj, Nandagopal Kaliappan

**Affiliations:** 1 Material Science Lab, Department of Prosthodontics, Saveetha Dental College and Hospitals, SIMATS, Saveetha University, Chennai, Tamilnadu India; 2 Department of Mechanical Engineering, Saveetha School of Engineering, SIMATS, Saveetha University, Chennai, Tamilnadu India; 3 Department of Periodontics, Saveetha Dental College and Hospitals, SIMATS, Saveetha University, Chennai, Tamilnadu India; 4 Department of Mechanical Engineering, Haramaya Institute of Technology, Haramaya University, Dire Dawa, Ethiopia

**Keywords:** Engineering, Mechanical engineering

## Abstract

The present study aims to examine the characteristics of a composite material composed of glass/madar fibers and porcelain particles, which are reinforced with epoxy. A compression molding technique achieves the fabrication of this composite. A comprehensive characterization was conducted by employing a mixture of analytical techniques, including X-ray Diffraction (XRD), mechanical testing, Scanning Electron Microscopy (SEM), Dynamic Mechanical Analysis (DMA), and Thermogravimetric Analysis (TGA). The composition of the composite was determined using X-ray diffraction (XRD) analysis, which demonstrated the successful integration of porcelain fillers. The material exhibited notable mechanical properties, rendering it appropriate for utilization in structural applications. The utilization of SEM facilitated the examination of the microstructure of the composite material, thereby providing a deeper understanding of the interactions between the fibers and the matrix. DMA results revealed the glass/madar composite contained 4.2% higher viscoelastic properties when the addition of porcelain filler, thermal stability was improved up to the maximum temperature of 357 °C. This study provided significant insights into the properties of a hybrid epoxy composite consisting of glass/madar fibers reinforced porcelain particles.

## Introduction

A polymer matrix composite (PMC) is a highly adaptable material that consists of a polymer resin matrix that is reinforced with fibers or particles. This particular category of composite materials has a distinctive amalgamation of lightweight properties and exceptional strength, rendering them indispensable in diverse sectors, including aircraft, automotive, and construction^1^. The selection of the polymer matrix and reinforcing material can be customized to meet individual application demands, leading to a diverse array of attainable qualities^2^. Polymer matrix composites are renowned for their exceptional resistance to corrosion, remarkable durability, and remarkable capacity to be shaped into intricate forms. These qualities render them a favored option for the production of lightweight, high-performance components across several industries^3^. Hybrid composites, which involve the integration of synthetic and natural fibers as reinforcement elements, exemplify a captivating amalgamation of contemporary technology with environmentally friendly resources^4^. These composite materials exploit the intrinsic mechanical properties of synthetic fibers, such as carbon or glass, which possess high strength and stiffness. At the same time, they also take advantage of the renewable and lightweight characteristics offered by natural fibers like flax, jute, or hemp. The combination of these factors leads to the development of composites that exhibit a commendable equilibrium in terms of strength, weight, and environmental conscientiousness^5^. Hybrid composites are extensively utilized in several industries, including automobile components, athletic goods, and construction materials, due to their commendable amalgamation of performance attributes and environmental sustainability, which are much esteemed^6^. The utilization of natural fiber composites is increasingly prevalent in various industries such as automotive, construction, and consumer goods, mostly attributed to their advantageous characteristics, including lightweight composition, favorable mechanical attributes, and sustainable nature^7^. These materials not only mitigate the dependence on finite resources but also provide prospects for improving the environmental efficacy of diverse products and applications^8^. Glass fiber composites are widely recognized for their excellent characteristics, rendering them indispensable in diverse applications. The materials commonly demonstrate a tensile strength that spans from 34 to 72 MPa, along with a high modulus of elasticity that normally ranges from 0.34 to 0.76 GPa^9^. In addition, these composite materials exhibit exceptional resilience to environmental influences, characterized by a relatively low coefficient of thermal expansion of around 5.5 × 10^−6^ per degree Celsius. In addition, glass fiber composites exhibit a comparatively modest density, typically ranging from 1.8 to 2.6 g per cubic centimeter (g/cm^3^), hence enhancing their inherent lightweight characteristics^10^. The combination of these characteristics renders glass fiber composites highly advantageous for a diverse array of structural and technical purposes. Madar fibers possess essential characteristics that render them appropriate for utilization in composite applications. Typically, the materials exhibit a tensile strength that falls within the range of 30 to 50 MPa, accompanied by a modulus of elasticity ranging from 0.3 to 0.6 GPa. These fibers possess inherent qualities of being widely available and environmentally friendly, with a very modest density ranging from 1.3 to 1.5 g/cm^3^^11^. Furthermore, the utilization of madar/jute fibers presents commendable thermal insulating characteristics and biodegradability, thereby aligning with the imperative of eco-consciousness and the necessity for lightweight composite materials^12^. The characteristics above provide madar fiber composites a desirable option for businesses that prioritize sustainable and moderately robust materials, including vehicle interiors, packaging, and building materials^13^. Porcelain fillers are frequently employed in epoxy composites to augment a range of qualities. Composite materials commonly demonstrate a notable level of compressive strength, typically falling within the range of 20 to 50 MPa^14^. This characteristic plays a significant role in enabling the composite to endure substantial loads and effectively withstand compression-induced deformations. Porcelain fillers are known for their exceptional electrical insulating characteristics, characterized by high dielectric strength, often falling within the range of 15 to 25 MV/m. Moreover, the thermal conductivity of porcelain-filled epoxy composites is relatively low, often ranging from 1.5 to 2.5 W/mK. This characteristic renders them well-suited for various applications that demand effective thermal insulation. The characteristics above render porcelain fillers highly advantageous in composite materials employed in optical, electronics, electrical insulation, and thermal management applications^15^. The storage modulus and loss modulus are fundamental mechanical characteristics of epoxy composites reinforced with a combination of glass and kenaf fibers. The storage modulus, which characterizes the capacity of the material to store elastic energy, typically lies within the range of 2000 to 4000 MPa for these composite materials. Conversely, the loss modulus, which signifies the material's capacity to dissipate energy during deformation, generally falls between the ranges of 100 to 300 MPa^16^. The aforementioned numbers serve as evidence that the hybrid composite possesses the ability to endure significant loads while still maintaining favorable damping properties. The amalgamation of characteristics exhibited by glass/kenaf fibers hybrid epoxy composites renders them well-suited for use in scenarios where achieving a harmonious equilibrium between rigidity and vibration attenuation is of utmost importance^17^. The damping factor, an essential mechanical characteristic of epoxy composites reinforced with glass/ramie fibers, typically falls within the range of 0.02 to 0.08. This parameter measures the capacity of the substance to disperse vibrational energy under the influence of dynamic stresses^18^. The composites exhibit a comparatively elevated damping factor, rendering them beneficial for applications that necessitate vibration control and energy dissipation. This is particularly relevant in the fabrication of automotive components and structures that are susceptible to dynamic loads^19^. X-ray diffraction (XRD) analysis is a technique commonly employed to elucidate the crystalline structure of composite materials. The resulting peak values, typically observed within the range of 10 to 30 degrees, offer valuable insights into the structural features of these materials^20^. TGA tests provide significant insights into the thermal stability of the composite, elucidating weight loss patterns and breakdown temperatures. As an illustration, the composite material may demonstrate an initial reduction in weight ranging from 5 to 10% at approximately 250 °C, attributable to the presence of moisture and volatile chemicals^21^. Scanning electron microscopy (SEM) possesses the capability to capture intricate microstructural features, such as interactions between fibers and matrices, with magnifications typically ranging from 100 × to 1000x. The collective examination of these evaluations provides a full comprehension of the structure, thermal characteristics, and microstructure of the composite, hence facilitating its design and optimization of performanc ^22^. When the addition of porcelain material in polymer composite can enhance the mechanical and thermal stability due to the unique properties of the filler, also it is highly resistant of wear, chemical, and corrosion, therefore when the fabrication of composite with these material can be used in automobile applications such as car dash panel, doors.

The main objective of this study is to produce composite laminates by the application of a compression molding technique, with the intention of examining the effects of altering the weight ratios of reinforcement materials (namely glass and madar) and filler material (porcelain) with epoxy matrix. This study aims to evaluate the influence of composite laminates by the implementation of analyses in several domains, including crystalline structure, mechanical properties, dynamic behavior, and thermal characteristics. Furthermore, this research incorporates a comprehensive morphological analysis in order to effectively identify and document the many failure modes present in the hybrid composite material under SEM analysis.

## Materials and experimental methods

### Materials used

This study complies with relevant institutional, national, and international guidelines and legislation. The composite material was fabricated, including synthetic glass fiber mats weighing 300 g per square meter and natural madar fiber obtained from Composite Mart Pvt. Ltd., located in New Delhi, India. The porcelain fillers, epoxy resin, and hardener (LY-556/HY-951) were procured from Janki Enterprises, a supplier based in Chennai, India. The thickness of each fiber mat was approximately 2 mm. The composite was fabricated using a compression molding technique. The technique above was employed in the creation of five unique laminates characterized by variations in both sequence and weight fractions. The laminates in question exhibit a composition consisting of a balanced mixture of 47.5% reinforcement materials, namely glass and madar fibers, and 5% filler material, specifically porcelain, within an 47.5% epoxy matrix.

**Figure 1:**
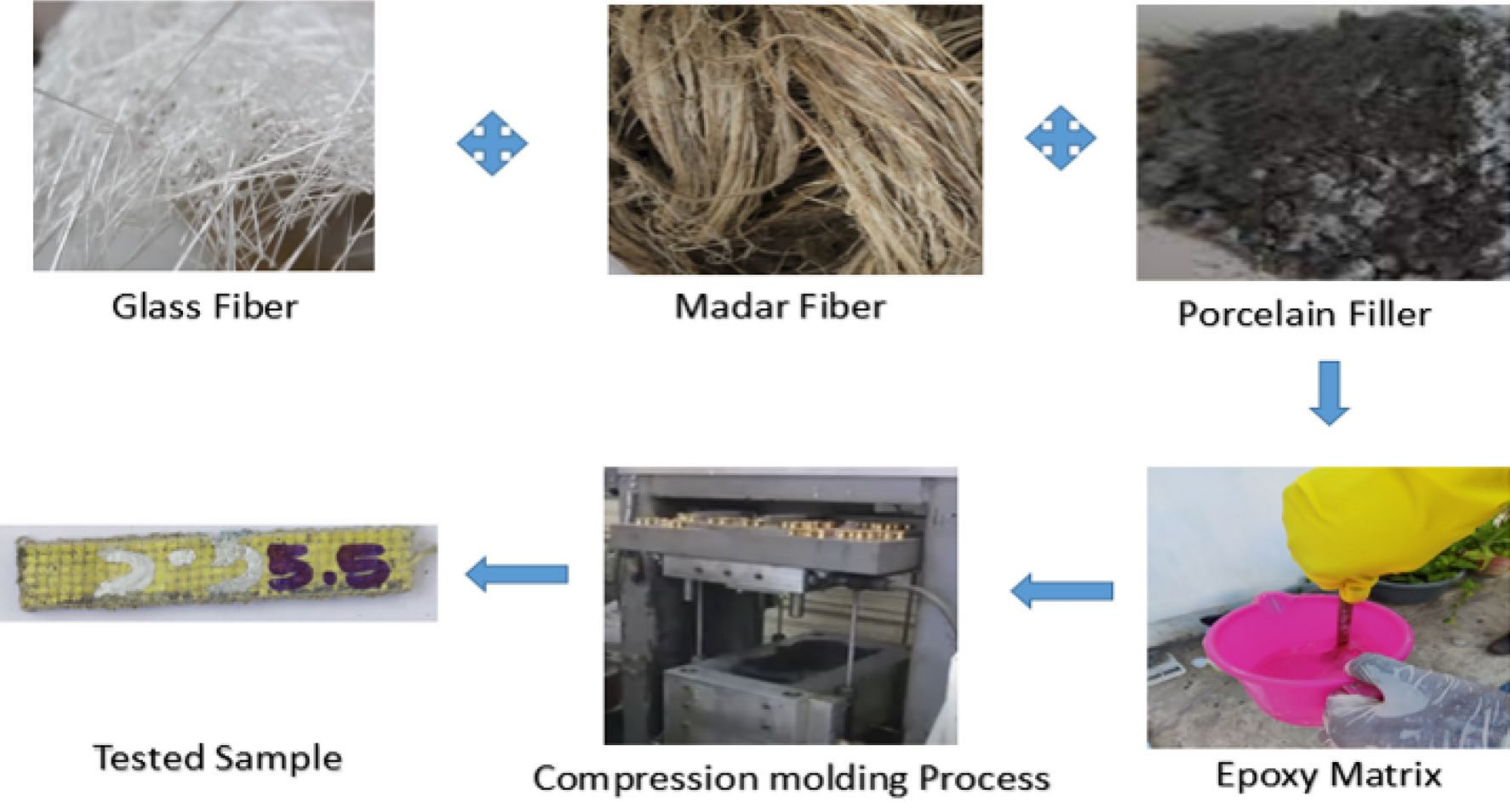
Fabrication Process of Hybrid Composite.

The compression molding technique is utilized in the production process of glass/madar fiber mat hybrid epoxy composites, encompassing a series of essential stages. In the first stage, glass fibers and madar fibers are fabricated and arranged in a predetermined configuration, typically in the shape of many layers (five layers), in order to achieve optimal dispersion within the composite materia ^23^. The epoxy resin is combined with porcelain fillers and other required additives and subsequently blended meticulously to get a uniform matrix. The fiber mats that have been manufactured are saturated with a mixture of epoxy resin in order to achieve a robust bonding. Compression molding is a manufacturing technique that involves the simultaneous application of pressure and heat. This process is commonly carried out at a temperature of 120 degrees Celsius and a pressure of 10 MPa in order to consolidate the layers of a composite material^24^. The procedure above enables the successful curing of epoxy resin, leading to the formation of a robust and cohesive composite material consisting of glass/madar fibers and epoxy. The identical methodology was employed in the production of the remaining four samples, each of which included a distinct arrangement of glass and madar fibers, are shown in Fig. 1. Table 1 presents the weight and Table 2 shows the weight fraction of the composite consisting of glass and madar fibers.

**Table 1:** Weight of materials used in hybrid composite.

Sample Code	Epoxy matrix in g	Porcelain filler in g	Glass fiber in g	Madar fiber in g
L0	200	0	200	0
L1	200	20	200	0
L2	200	20	150	50
L3	200	20	100	100
L4	200	20	50	150
L5	200	20	0	200
L6	200	0	0	200

**Table 2:** Weight ratio of materials used in hybrid composite.

Sample Code	Epoxy matrix in %	Porcelain filler in %	Glass fiber in %	Madar fiber in %
L0	50	0	50	0
L1	47.5	5	47.5	0
L2	47.5	5	35.5	12
L3	47.5	5	23.75	23.75
L4	47.5	5	12	35.5
L5	47.5	5	0	47.5
L6	50	0	0	50

### Experimental testing

A thorough array of investigations was conducted to assess the characteristics and performance of the hybrid epoxy composite during experimental testing. The utilization of X Pert—X-ray Diffraction (XRD) was employed as per ASTM E0915 standard to investigate the crystalline structure of the composite material 500 mg sample was taken, thereby offering valuable atomic arrangement and the various phases that may be present. Mechanical testing was conducted to evaluate different features such as tensile test by using Universal Testing Machine at 100 kN load condition with ASTM D638 standard, flexural test was conducted by 3-point bending analysis with ASTM D790 standard, and the Izod impact test was conducted by ASTM D256 standrd with the dimention of 75 × 13 × 5, in order to determine the mechanical qualities and structural integrity of the material. The utilization of JEOL SEM-2100F facilitated a comprehensive analysis of the microstructure, enabling the observation of surface morphology and the interactions between the fibers and matrix in the composite material. The viscoelastic behavior of the material was investigated through the utilization of Rheometric Scientific SR-5000 dynamic mechanical analyzer (DMA), the data were collected from 30 to 180 °C at a scanning rate of 6 °C/min. which involved the examination of its stiffness and damping properties across various temperatures and frequencies. The thermal stability of the composite was assessed using TA instrument Q5000 thermogravimetric analyzer (TGA), with 10 mg of samples was heated from 30 to 400 °C in a nitrogen atmosphere which involved measuring the loss in weight as the temperature increased^25^. This analysis provided vital insights into the composite's capacity to withstand degradation caused by elevated temperatures.

## Results and discussion

### XRD analysis of glass/madar fibers hybrid composite

**Figure 2:**
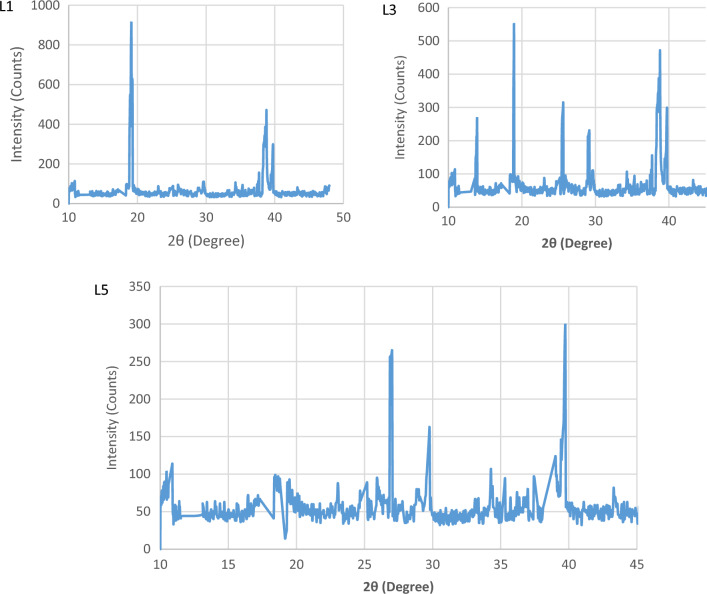
XRD curve of glass/madar composite sample.

X-ray diffraction (XRD) study was performed on a newly developed hybrid epoxy composite sample L1 consisting of glass/madar fibers and porcelain filler in order to obtain a deeper understanding of its crystalline structure. The X-ray diffraction (XRD) analysis exhibited well-defined diffraction peaks at specified angles, suggesting the existence of crystalline phases inside the composite material. The primary diffraction peak was observed at an angle of 2θ = 18.4°, indicating the presence of a crystalline phase in the porcelain filler. This phase was identified as α-Al2O3, which is alumina^26^. Furthermore, an attenuated peak with a 2θ value of 38.1° was detected, indicating the existence of crystalline domains related to the epoxy matrix. The magnitude of this peak, however, exhibited a notable decrease in comparison to the porcelain peak, suggesting that the epoxy in the composite is primarily amorphous. The crystalline values provide significant insights into the composition and crystalline content of the hybrid composite, hence enhancing comprehension of its structural features. The incorporation of filler material was employed to augment the composite's crystalline area, which accounted for 38.4% of the total composition, while the remaining 62.6% comprised the amorphous region. This effect was observed in the presence of natural madar fiber. The incorporation of a greater proportion of an amorphous region in this hybrid composite has the potential to decrease its wettability and enhance its mechanical capabilities. Figure 2 shows the XRD result of sample (L1, L3, & L5) hybrid composite.

In a separate study, a prominent peak was observed at a 2θ value of 20.3°, indicating the presence of the crystalline cellulose structure within sisal fibers. An additional, prominent peak was seen at an angle of 2θ = 25.9°, indicating the presence of an amorphous structure inside the epoxy resin matrix. Significantly, the peak intensity of the sisal fiber was notably greater in comparison to that of the epoxy, indicating the prevalence of cellulose crystallinity within the composite. This underscores the substantial existence of sisal fibers and the amorphous characteristics of the epoxy matrix ^27^.

### Mechanical properties of glass/madar fibers hybrid composite

**Figure 3:**
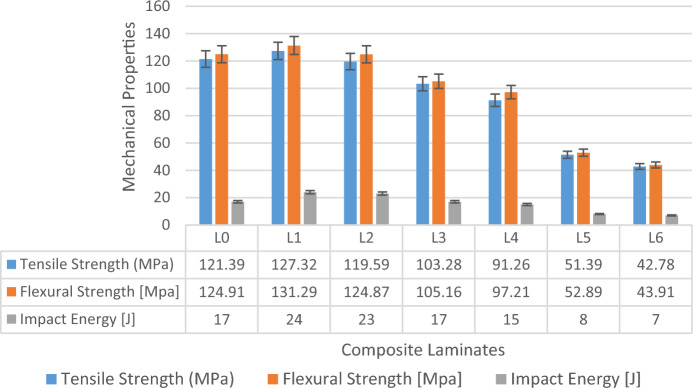
Mechanical Properties of glass/madar fibers composite.

The investigation of the tensile strength of the hybrid epoxy composite consisting of glass/madar fibers and porcelain fillers shows noteworthy mechanical characteristics. A notable observation was made regarding the greatest tensile strength displayed by sample L1, which was 127.32 MPa. The capacity of the composite material to endure axial pressures under stress, rendering it a resilient substance suitable for diverse engineering purposes. The tensile strength of the composite is improved with the addition of glass and madar fibers, as these fibers serve as a kind of reinforcement. Additionally, the inclusion of porcelain fillers contributes to the overall stiffness of the composite^28^. Figure 3 shows the mechanical properties of hybrid composite.

The hybrid epoxy composite, consisting of glass/madar fibers and porcelain fillers, demonstrated significant flexural and impact strength qualities, hence validating its appropriateness for a wide range of technical applications. During the flexural tests, the composite material exhibited a measured flexural strength of around 131.29 MPa, indicating its ability to resist bending and deformation when subjected to external loads. In addition, an evaluation was conducted to determine the impact strength of the composite, resulting in a measured value of 24 kJ/m^2^. The impact energy value of this glass/madar fiber composite serves as an indicator of the composite material's capacity to absorb energy and withstand fracture under the influence of abrupt impact loads. This attribute holds significant value in contexts where the ability to withstand impact is of utmost importance, such as in the realm of manufacturing machinery and protective attire^29^.

### SEM morphology of fractured surface on hybrid composite

**Figure 4:**
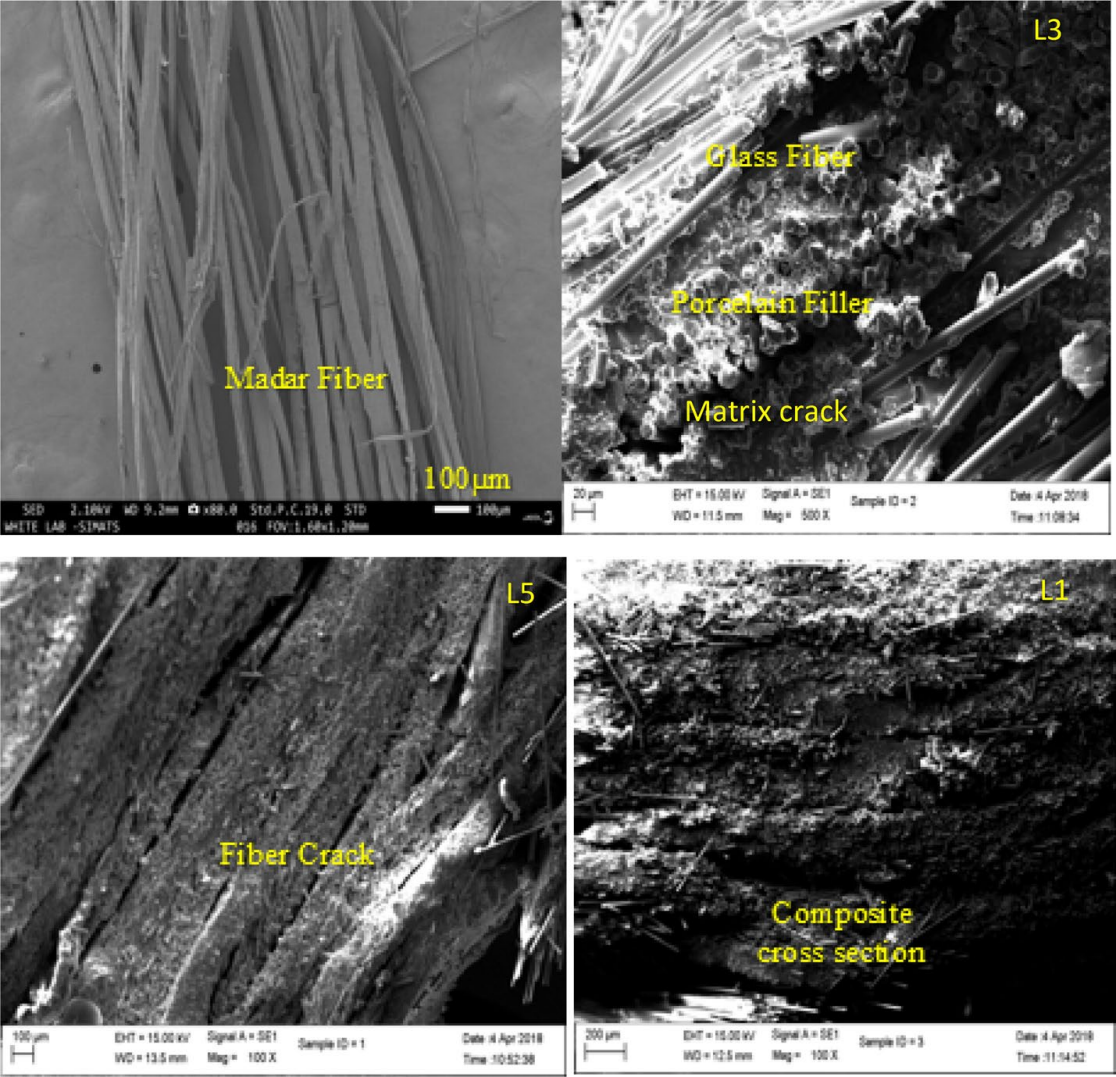
SEM Micrographs of glass/madar hybrid composite.

The morphological characteristics of the glass/madar fibers with porcelain fillings hybrid epoxy composite were examined using Scanning Electron Microscopy (SEM) analysis of the broken surfaces resulting from tensile testing on this hybrid composite. This study yielded useful insights into the composite's morphological properties^30^. During the process of conducting the tensile test, the SEM images provided visual evidence of a discernible pattern characterized by the occurrence of fiber pull-out and elongation, accompanied by notable interactions between the fibers and the surrounding matrix material. The observed broken surface exhibited a coarse topography characterized by several micro-cracks, fiber breakage, and matrix cracks are more in sample L3 and L5 compared sample L1 which are suggesting the composite material's capacity for efficient stress distribution. The observed surface roughness and the occurrence of fiber bridging exhibited a correlation with improved mechanical characteristics. Figure 4 shows the micrograph of the hybrid composite.

The observed fractured surface exhibited indications of localized damage, characterized by the presence of micro spaces and debonding occurring between the fibers and matrix. The SEM images provided more insight into the energy-absorbing mechanisms observed in the composite material, including crack deflection and branching^31^.

### DMA analysis of glass/madar fibers hybrid composite

**Figure 5:**
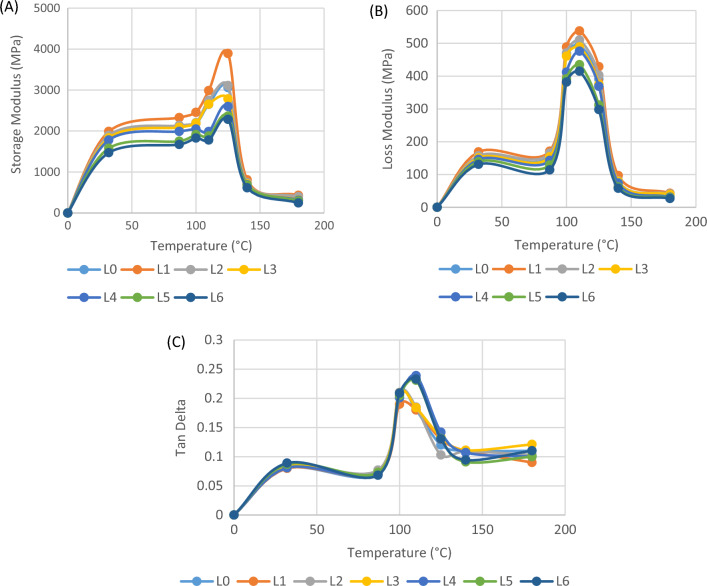
Visco elastic behaviors of glass/madar fibers composite.

Dynamic mechanical analysis (DMA) is a widely employed method for the evaluation of viscoelastic characteristics in polymers and composites. This methodology enables the determination of key parameters like the storage modulus (E'), loss modulus (E''), and tan delta (damping factor). The storage modulus of the hybrid epoxy composite consisting of glass/madar fibers and porcelain fillers was investigated using Dynamic Mechanical Analysis (DMA) at various temperatures^32^. The temperature dependence of the storage modulus, which characterizes the stiffness and capacity of a material to retain elastic energy, was observed to be significant. The storage modulus of the composite material was evaluated within a temperature range of 32 to 180 °C, maximum yielding values ranging from 431 to 3893 MPa in sample L1 and the minimum E was observed in sample L6 is 245 to 2285 MPa. These results indicate the exceptional stiffness and load-bearing capabilities of the composite. The storage modulus of the hybrid composite increases at temperatures up to 115 °C, indicating an increase in molecular mobility and free volume. However, once it surpasses the glass transition temperature, the storage modulus of the composite laminates suddenly decreases at 132 °C, the hybrid composites exhibit the lowest storage modulus. With the rise in temperature, there was a gradual decrease observed in the storage modulus, ultimately reaching a value of around 810 MPa at a temperature of 140 °C. The observed decrease in storage modulus as temperature increases suggests a change from a glassy to a more rubbery state in the composite, which is a characteristic behavior of epoxy materials. The loss modulus, which is a measure of the energy dissipation resulting from internal friction within a material, displayed unique behavior as the temperature varied. The loss modulus of the composite material was determined within the temperature range of 32 to 180 °C, with values ranging from roughly 43 to 438 MPa. With the rise in temperature, there was an observed increase in the loss modulus, which eventually reached a value of around 81 MPa at a temperature of 140 °C. The observed rise in loss modulus with increasing temperature suggests a shift from a glassy to a viscoelastic state, indicating the composite's capacity to absorb energy and withstand deformation under high temperatures. This indicates that the inclusion of fiber reinforcement has resulted in an increase in the friction between the matrix and fibers, hence enhancing the dissipation of energy. This finding has also been observed by other researchers^32^. The damping factor, denoted as tan delta (δ), quantifies the capacity of a substance to dissipate energy in response to variations in temperature. This quantity holds significant importance in the assessment of a material's viscoelastic properties. The tan delta of the composite material was determined to be roughly 0.08 to 0.13 within the temperature range of 32 to 180 °C, suggesting a modest degree of energy dissipation. As the temperature rose, there was a noticeable upward trend in the tan delta values, with a peak of around 0.05 seen at a temperature of 140 °C. The increased limitations on the shapeless state result in a more pronounced or wider glass transition behavior, with the largest peak width observed in composites with a larger glass fiber loading. The observed increase in tan delta with temperature indicates a shift from predominantly elastic behavior to a more viscoelastic behavior in the composite material, indicating its improved ability to absorb energy at higher temperatures. Figures 5A–C show the storage modulus, loss modulus, and damping factor of the glass/madar fibers hybrid composite.

Conversely, the loss modulus (E") or dynamic loss modulus characterizes the viscoelastic behavior of a substance and is indicative of the dissipation of thermal energy within the sample. This variable is commonly associated with the phenomenon of internal friction in the material^33^. The primary factors influencing it are the molecular configurations, variations, movements, and phase transitions. The complex modulus (E*) is a word used to characterize the resulting value of loss modulus and storage modulus, which collectively represent a material's resistance to deformation. The glass transition temperature (Tg) refers to the temperature at which a thermosetting and amorphous polymer experiences a change from a hard molecular arrangement to a more flexible, rubbery state^34^. It is important to acknowledge that the glass transition temperature (Tg) should not be conflated with the melting temperature (Tm), as the latter is the temperature at which a material begins to transition from a solid to a liquid state, often observed in polymers with a crystalline arrangement. The glass transition temperature (Tg) exhibits a strong correlation with the degree of cross-linking in a polymer^28^.In the case of thermosets, a greater cross-link density imposes limitations on molecular movements, leading to an increased energy need for facilitating segmental mobility during the glass transition. As a result, this behavior leads to an increased modulus, decreased damping factor, and elevated glass transition temperature (Tg). The impact of cross-link density is discernible in the rubbery and transition phases of a material but not in the glassy region^4^. Therefore, it can be observed that the modulus values of lightly and heavily cross-linked polymers exhibit similarity inside the glassy region of the dynamic mechanical analysis (DMA) curve. On the other hand, it is worth noting that in the rubbery and transition portions of the curve, a strongly cross-linked polymer will have significantly elevated modulus values, indicating a more compact molecular structure and increased rigidity.

### TGA analysis of glass/madar fibers hybrid composite

**Figure 6:**
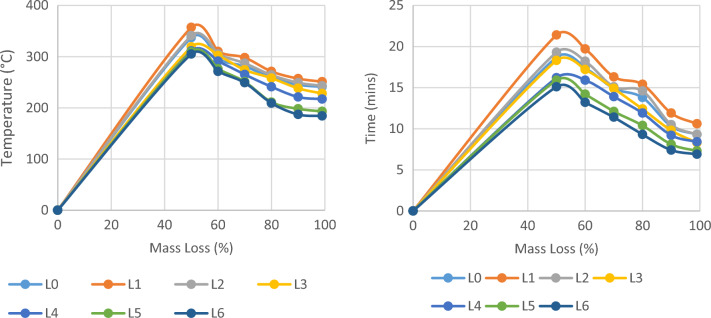
Thermal stability of glass/madar fibers hybrid composite.

Thermal stability and decomposition behavior of the hybrid epoxy composite, consisting of glass/madar fibers and porcelain fillers, were evaluated through the implementation of Thermogravimetric Analysis (TGA). The thermogravimetric analysis (TGA) curve provided significant insights into the material's behavior when subjected to high temperatures^8^. At the outset, the composite material experienced a slight reduction in weight, approximately 2%, within the temperature range of 130 to 190 °C. This phenomenon can be attributed to the elimination of moisture and volatile substances present at lower temperatures. The primary disintegration stage took place within the temperature range of 200 to 300 °C, resulting in a notable reduction in weight of around 45% for the composite material. The observed loss can be mostly attributed to the degradation of the epoxy matrix and the combustion of organic constituents included in the composite material. In a separate study, the thermal stability of neat epoxy matrix and sisal fiber composites was investigated, whereby it was observed that the early mass loss occurred at a temperature of 110 °C for neat epoxy matrix. When the addition of sisal fiber can improve the thermal stability with the primary mass loss occurred at 198 °C^7^. Hence, the incorporation of synthetic glass fiber has the potential to improve the thermal stability of composite materials. At temperatures exceeding 400 °C, an enduring residual mass of roughly 23% was detected, indicating the potential existence of inorganic fillers such as porcelain and reinforcing fibers^25^. The TGA results offered valuable insights into the thermal stability of the composite, including breakdown temperatures and the degree of weight loss at various stages. This information is crucial for selecting the composite for applications that involve exposure to high temperatures. Figure 6 shows the thermal stability of the hybrid composite.

## Conclusion

In summary, the thorough examination of the hybrid epoxy composite consisting of glass/madar fibers reinforced porcelain particles has yielded a number of significant discoveries. The successful integration of alumina (α-Al2O3) from porcelain fillers into the composite structure was confirmed through the utilization of X-ray Diffraction (XRD) analysis. The mechanical testing results revealed notable tensile strength (127.32 MPa), flexural strength (131.39 MPa), and impact resistance (24 kJ/m^2^), indicating that the material is well-suited for load-bearing applications. The utilization of Scanning Electron Microscopy (SEM) facilitated the examination of the morphological characteristics of the composite material, thereby emphasizing the interactions between the fibers and the matrix, as well as the mechanisms involved in fracture. The temperature-dependent behavior of Dynamic Mechanical Analysis (DMA) was observed, wherein the storage modulus exhibited a change from roughly 1989 MPa at 32 °C to approximately 3893 MPa at a temperature of 125 °C. This observation suggests a transition from a vitreous to a viscoelastic state in the substance. The damping factor, also known as tan delta, exhibited a rise from around 0.08 to 0.13 across the identical temperature range, indicating a shift towards a viscoelastic response. The thermal stability of the material was assessed by Thermogravimetric Analysis (TGA), revealing a notable residual mass (23%) observed at temperatures over 400 °C. This observation implies the likely inclusion of inorganic fillers inside the material. The findings of this research offer significant knowledge and understanding that can be applied to many engineering and industrial applications.

## Data Availability

The datasets used and/or analyzed during the current study available from the corresponding author on reasonable request.
